# Good practice in lactation counseling for Ukrainian refugee mothers to ensure the health and mental benefits of breastfeeding – an observational study

**DOI:** 10.1007/s00737-024-01538-x

**Published:** 2024-12-06

**Authors:** Magdalena Babiszewska-Aksamit, Agnieszka Bzikowska-Jura, Anna Kotlińska, Agata Aduła, Agnieszka Chrobak, Justyna Domosud, Izabela Drążkowska, Paulina Gaweł, Artur Jakimiuk, Józefa Kołodziej, Barbara Królak-Olejnik, Katarzyna Lisak-Gurba, Katarzyna Mozdyniewicz, Aleksandra Mołas, Agnieszka Piątkowska, Elena Sinkiewicz-Darol, Aleksandra Wesołowska

**Affiliations:** 1 Laboratory of Human Milk and Lactation Research, Department of Medical Biology, Medical University of Warsaw, Warsaw, Poland; 2 Human Milk Bank Foundation, Warsaw, Poland; 3 Medical Simulation Center, Andrzej Frycz Modrzewski Academy in Krakow, Krakow, Poland; 4 Mother and Child Health Center in Zielona Góra, Zielona Góra, Poland; 5 Falkiewicz Specialist Hospital in Wroclaw, Wroclaw, Poland; 6 Independent Public Clinical Hospital, No. 1 in Lublin, Lublin, Poland; 7 Department of Neonatology, Medical University of Gdańsk, Gdańsk, Poland; 8 Department of Neonatology Medical University in Wrocław, University Hospital in Wrocław, Wrocław, Poland; 9 State Medical Institute of the Ministry of Internal Affairs and Administration in Warsaw, Warsaw, Poland; 10 Pro-Familia Specialist Hospital in Rzeszów, Rzeszów, Poland; 11 Chopin University Clinical Hospital in Rzeszów, Rzeszów, Poland; 12 Ujastek Obstetrics and Gynecology Hospital in Krakow, Krakow, Poland; 13 Institute of the Polish Mother’s Health Center in Łódź, Łódź, Poland; 14 Ludwik Rydygier Provincial Polyclinical Hospital, Human Milk Bank, Torun, Poland; 15 Department of Physiology and Toxicology, Faculty of Biological Sciences, Kazimierz Wielki University, Bydgoszcz, Poland

**Keywords:** Breastfeeding, Mental health, Refugee crisis, Lactation support

## Abstract

**Purpose:**

The study presents a cross-sectional analysis of the population of Ukrainian women who received maternity care in 11 Polish hospitals.

**Methods:**

Multidirectional lactation counseling was implemented from March to November 2023 by the Human Milk Bank Foundation in cooperation with UNICEF Refugee Response Office in Poland. Medical data were collected using questionnaires prepared for the study. When the mother indicated that she had mental problems or the health care personnel spotted problems, she was she referred to a psychologist for diagnostics, who used questionnaires appropriate to the patient’s situation such as: Patient Health Questionnaire-9 for depression assessment and Generalized Anxiety Disorder Questionnaire-7 for anxiety assessment.

**Results:**

In total, 1203 consultations were carried out, of which 542 were lactation counseling, 305 - were physiotherapy, 227 - were psychological, and 129 - were with speech-language pathologists. Two hours of skin-to-skin contact (SSC) and latching on the breast within the 1st hour postpartum occurred in more than half of the participants. In the rest of the population, shortened or lack of SSC was associated with breastfeeding delayed by at least one day. 53% of the population required lactation counseling, of which 242 were one-time visits. Continued support was 98% effective in achieving breastfeeding goals. 167 mothers required psychological support, of which only 53 women continued treatment beyond one-time counseling. Only about 1% of women experienced mental disorders based on a psychological interview.

**Conclusion:**

The use of lactation counseling more often than psychological counseling by refugee mothers could be related to the beneficial effects of breastfeeding on maternal well-being through hormonal self-regulation and empowerment as a mother. However, focused research is needed on the impact of breastfeeding on the mental health of refugee women.

**Supplementary Information:**

The online version contains supplementary material available at 10.1007/s00737-024-01538-x.

## Introduction

The perinatal period (from pregnancy to the first year postpartum) is very vulnerable in women’s lives and has been linked to the development of mental health disorders (Andersen et al. 2012; Fawcett et al. 2019). Although the perinatal period itself poses a risk of occurrence of mental health problems, refugee status is often associated with pre-migration experiences, socioeconomic disadvantage, and difficulties adapting to host cultures (Li and Browne 2000; Bhui et al. 2003; O’Mahony 2005) and lack of support, further increases the risk of serious mental health issues such as postpartum depression, (Falah-Hassani et al. 2015; Mechakra-Tahiri et al. 2007).

In a crisis, such as a natural disaster, pandemic, or war, the safest and the best food for newborns and infants is undoubtedly human milk (HM). The world’s largest international organizations, such as UNICEF, and WHO, emphasize in their recommendations (Summers and Bilukha 2018; WHO 2004; UNHCR 2018) that breastfeeding significantly reduces the risk of illness and death of infants in emergencies. HM is not only a perfectly balanced food for the infant that satisfies all the nutritional needs of the child but also protects against infections and other diseases (Ballard and Morrow 2013; Aleksaeev 2021). It can be strongly emphasized that in crises, mother’s milk saves children’s health and lives. Studies show that if breastfeeding was widespread among mothers, 823,000 deaths of children under the age of 5 years could be prevented per year worldwide (Victora et al. 2016). Unfortunately, studies conducted in Iraq Ukraine and Croatia have shown that breastfeeding rates during wartime drastically declined with 4–7% fewer mothers breastfeeding compared to the times of peace (Brink 2019) due to the lack of support from family and medical personnel and the level of stress experienced by the mother during wartime (Diwakar et al. 2019; Guerrero Serdan 2009; Summers and Bilukha 2018; Zakanj et al. 2000). Therefore, if there are no medical contraindications to breastfeeding, it is crucial to support mothers in this decision and effort (Summers and Bilukha 2018; WHO 2004; UNHCR 2018).

Many studies have also shown that breastfeeding has the potential to directly decrease the symptoms and protect mothers against postpartum depression (Figueiredo et al. 2014), and anxiety (Groer and Davis 2006). Moreover, there is a strong correlation between lactation and reduced stress responses in mothers (Mezzacappa and Katkin 2002) thanks to decreased secretion of hormones involved in the stress response such as cortisol and adrenocorticotropic hormone (ACTH) (Altemus et al. 1995; Amico et al. 1994; Handlin et al. 2009). Therefore, since breastfeeding has been shown to limit the activation of the hypothalamus-pituitary-adrenal (HPA) axis and secretion of glucocorticoids, ultimately reduces symptoms of postpartum depression (Altemus et al. 1995; Amico et al. 1994; Groer and Davis 2006). Moreover longer durations of skin-to-skin contact led to reduced levels of maternal cortisol, therefore limiting the symptoms of postpartum depression and anxiety (Handlin et al. 2009). What is more, breastfeeding, threw improvement of maternal self-efficiency (Field et al. 2002; Haga et al. 2012) and positive mother-infant relationship, induced by the infant’s touch of the mother’s nipple during the first four days after birth, impacts maternal well-being and may alter maternal neuroendocrine function and helps in reducing maternal depression (Widström et al. 1990). Research has also shown that not engaging in breastfeeding may increase the risk of postpartum depressive symptoms (Bilgin and Karabayır, 2024; Cerceo et al. 2024).

After the outbreak of the war in Ukraine on the 24th of February 2022, within the first three months of the war in Ukraine, 4.479 million people came to Poland. The largest group of refugees was women of reproductive age (17–49 years old) − 560,450 and children under 2 years of age − 60,000. There were no refugee camps in Poland, but there were reception and temporary residence points for displaced persons. Around 77% of Poles were involved in helping refugees from Ukraine in the early months of the war and most of them hosted one or more refugees in their homes (Baszczak et al. 2022). Over 6621 Ukrainian children were born in Poland during the first and half year of the conflict, most in the Mazovia region (*n* = 1220) and several hundred each in the main cities in Southern and Western Poland (data from National Health System).

Before the emergencies occurred, the rate of exclusive breastfeeding for up to 6 months in the Ukrainian population was not optimal (Summers and Bilukha 2018). According to the data from the Government Statistics Service in Ukraine in 2013, the early initiation of breastfeeding (EIBF) within 1 h of birth was 65.7%. The rate of exclusive breastfeeding below 6 months was only 19.7%. At the same time, 51.6% of children in this age were mixed-fed with a large breastfeeding component. According to the State Statistics of Ukraine (personal communication) in 2013 continuation of breastfeeding at one and two years of age concerned only 37.9% and 22% of children respectively. However, Ukrainian breastfeeding support experts claim that the rate of exclusive breastfeeding below 6 months of age is 56%, and 25% of children receive HM for up to one year (Romanenko et al. 2023). All Ukrainian refugees in Poland were provided with a full package of maternity and primary health care. Unfortunately, highly specialized lactation counseling provided by qualified personnel with an IBCLC certificate CDL (the Polish equivalent of IBCLC) is not reimbursed by the National Health Fund in Poland, and access to psychological counseling is very limited.

In this regard, the UNICEF Refugee Response Office in Poland has established a multi-directional lactation support program in 11 hospitals located in the 10 largest cities in the country. This project aimed to improve perinatal care for women experiencing the refugee crisis by expanding the scope of services provided by Polish hospitals to include hard-to-find specialist counseling, including lactation and psychological support. We expected that by providing multidimensional perinatal care, more Ukrainian mothers in the refugee crisis will decide to breastfeed their children, which in turn will improve their mental health and reduce the negative impact of stress associated with resettlement. A recent systematic review and meta-analysis examining the relationship between breastfeeding and maternal mental health highlighted a significant need for further research in this area due to the limited availability of data (Bugaeva et al. 2023). In this article, we summarized how multi-directional lactation assistance influenced the overall breastfeeding practices of Ukrainian refugee mothers and their well-being.

## Materials and methods

The study group consisted of 683 women. The inclusion criteria involved refugee status and having a child under 24 months of age. Almost all mothers 95.8%) (*n* = 654) were in the first stage of lactation (0–4 weeks postpartum), 3.7% (*n* = 25) had children aged 2–4 months, and 0.3% (*n* = 20) had children older than 6 months but not older than a year.

Between March 15, 2023 and November 30, 2023, refugee mothers from Ukraine were provided with support from lactation consultants, speech-language pathologists^1^, physiotherapists and psychologists in the 11 hospitals in Poland that were participating in the governmental program Pro-Life Program aimed to increase access to HM for vulnerable infants, including the provision of donor human milk (Appendix 1). We assessed postpartum hospital practices that support breastfeeding and bonding, the need for counseling by type, with particular attention to lactation and psychological counseling, how the baby was fed while the mother benefited from support, and the occurrence of serious mental health disorders in the mother. The implementation of the UNICEF project in 11 hospitals involved not only an increase in the scope of staff responsibilities but also the possibility of hiring new people, e.g. two midwives from Ukraine.

All women participating in the study completed a qualifying questionnaire, which included information on obstetric history, condition of the newborn/infant after delivery, child’s feeding in the hospital and after discharge, and postpartum good practices supporting exclusive breastfeeding. All surveys were completed by medical staff based on face-to-face interviews with mothers staying in maternity wards or retrospectively by respondents in hospitals participating in the project. Each participant received a unique ID number to anonymize the data and signed consent to participate in the project. At the end of the visit, the lactation consultant could refer the mother for another lactation consultation and/or the first consultation with a speech-language pathologist /pediatric physiotherapist/psychologist. All mothers have been recruited in one of the 11 hospitals implementing the UNICEF project could take advantage of 5 types of consultations free of charge (lactation, physiotherapy, speech therapy, neonatology, and psychology), which lasted an hour. Specialists in selected centers had at their disposal a device that translated in real-time into Polish from Ukrainian and vice versa. If the mother or specialist expressed such a need, she could make multiple appointments with a given specialist. Mothers could seek advice from a selected specialist when they expressed such a need, when they reported a problem with breastfeeding or psychological problems to the medical staff, or when the medical staff observed disturbing symptoms in the mother’s behavior during the recruitment visit. Also, when the problem regarding breastfeeding was very complicated, mothers were often referred for several consultations including a visit to a lactation consultant. Each lactation consultation was conducted by a certified lactation consultant (IBCLC or equivalent CDL certificate). Leaflets prepared by Human Milk Bank Foundation in Ukrainian and Polish about breastfeeding, the advantages of this way of feeding children, and the most common problems associated with it were distributed in maternity wards. All information about the project and hospitals participating in the UNICEF project were also available on the project’s website.

Psychologists used mental health disorder assessment questionnaires appropriate to the situation of selected patients who were referred for psychological counselling. For example, depression was assessed using the PHQ-9 (Patient Health Questionnaire), while anxiety was evaluated using the GDA-7 (Generalized Anxiety Disorder Questionnaire).

The Bioethics Committee at Warsaw Medical University accepted the information on the retrospective study and made no concerns (AKBE/332/2023).

## Results

### Characteristics of the study group

We examined 683 refugee mothers from Ukraine who gave birth to 697 babies (14 twins were born). The mean age of the recruited Ukrainian mothers was 30 ± 5.3 years. Women who were primiparous predominated (*n* = 366; 54%), while 17% of the studied mothers (*n* = 117) had at least 2 children. Most of the women gave birth vaginally (*n* = 447, 65,5%), whereas planned cesarean section was performed in 21% (*n* = 145) of the mothers. Most of the children (*n* = 580; 85%) were born on term (> 37 0/7 weeks of gestation), in turn, 11% (*n* = 78) were born before 33 0/7 weeks of gestation. In 93% of cases (*n* = 632), the child’s condition was good (Apgar scale: 8 and more). Most of the children, whose mothers were enrolled to the project, were newborns (*n* = 654; 96%) (Table 1).

**Table 1:** Descriptives of the study group

		N (%)	Mean (SD)	Min - max
Maternal age			29,6 (5,29) years	18–46 years
Gestational age			38,6 (2,37) weeks	23–42 weeks
Mode of delivery	CC	145 (21%)		
	Vaginal	447 (65,5%)		
	Vaginal finished with CC	91 (13,5%)		
Apgar scale	0–3	6 (1,5%)		
	4–7	40 (6%)		
	8–10	632 (92,5%)		
Infants age	0–1 month	652 (95,5%)		
	2–6 months	27 (3,9%)		
	7–24 months	4 (0,6%)		

## Demand for postpartum counseling

**Fig. 1:**
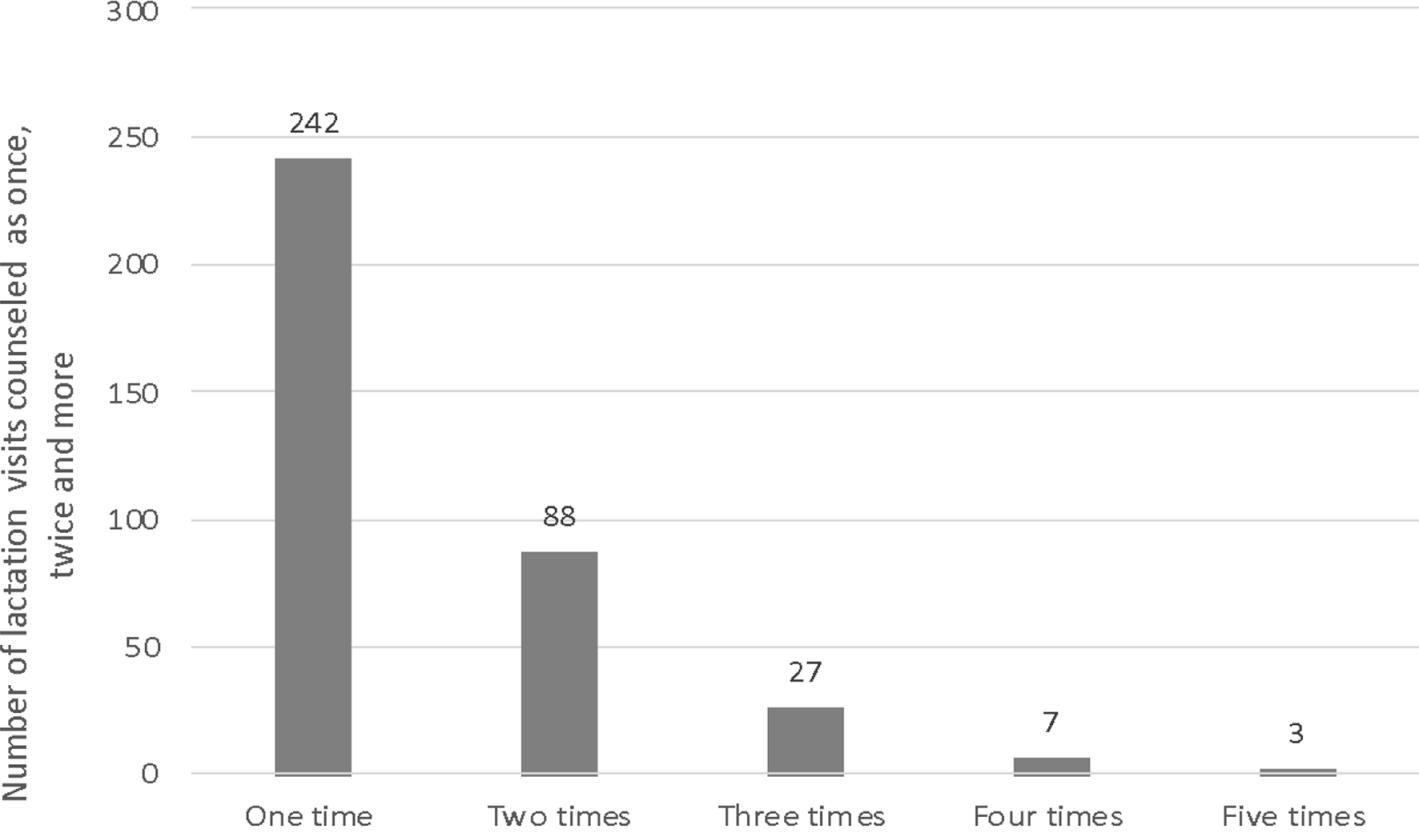
Number of lactation visits given once and more

All Ukrainian mothers (*n* = 683) participating in the project had unlimited access to health care in the field of lactation, physiotherapy, and speech-language pathologist therapy, with the exception of psychologists, due to the shortage of specialists in three hospitals. A total of 1203 one-hour specialist consultations took place, including 542 lactation visits, 305 physiotherapy visits, 227 psychological visits and 129 visits with a speech therapist. Most of the mothers (*n* = 367; 54%) required lactation support. Of all lactation counseling provided (*n* = 542), 452 attended one hour, 84 attended two hours, and 2 attended three hours visits. Most of the lactation counseling was provided on a one-time basis (*n* = 242; 66%), but 125 lactation-counseling visits took place as follow-up visits (Fig. 1).

**Fig. 2:**
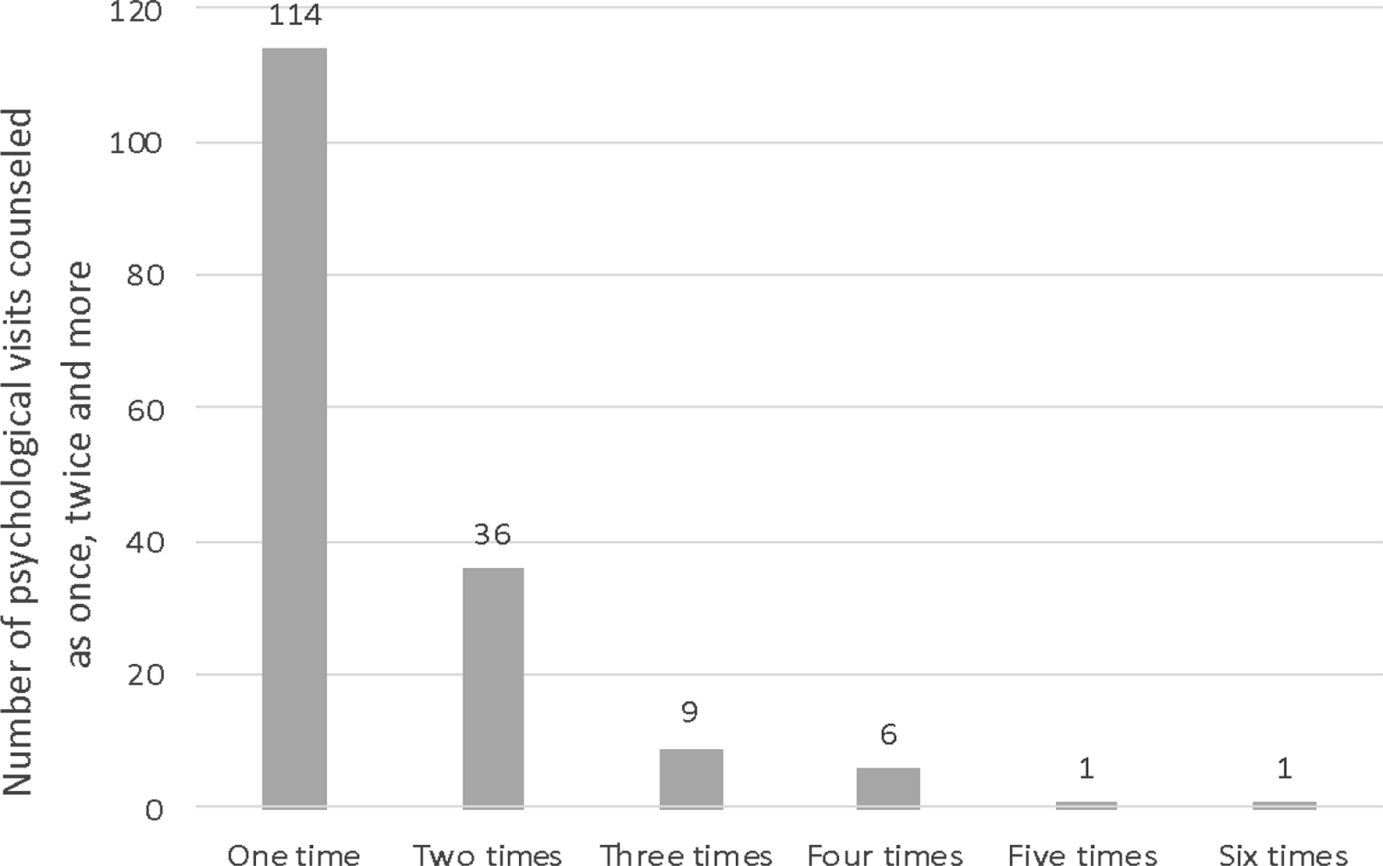
Number of psychological visits given once and more

Almost one in four women (*n* = 167; 24%) had a consultation with psychologists. Among the instances of psychological counseling, 68% (*n* = 114) consisted of a single visit, while only 32% (*n* = 53) involved ongoing psychological support beyond an initial session (Fig. 2).

## Postpartum care

**Fig. 3:**
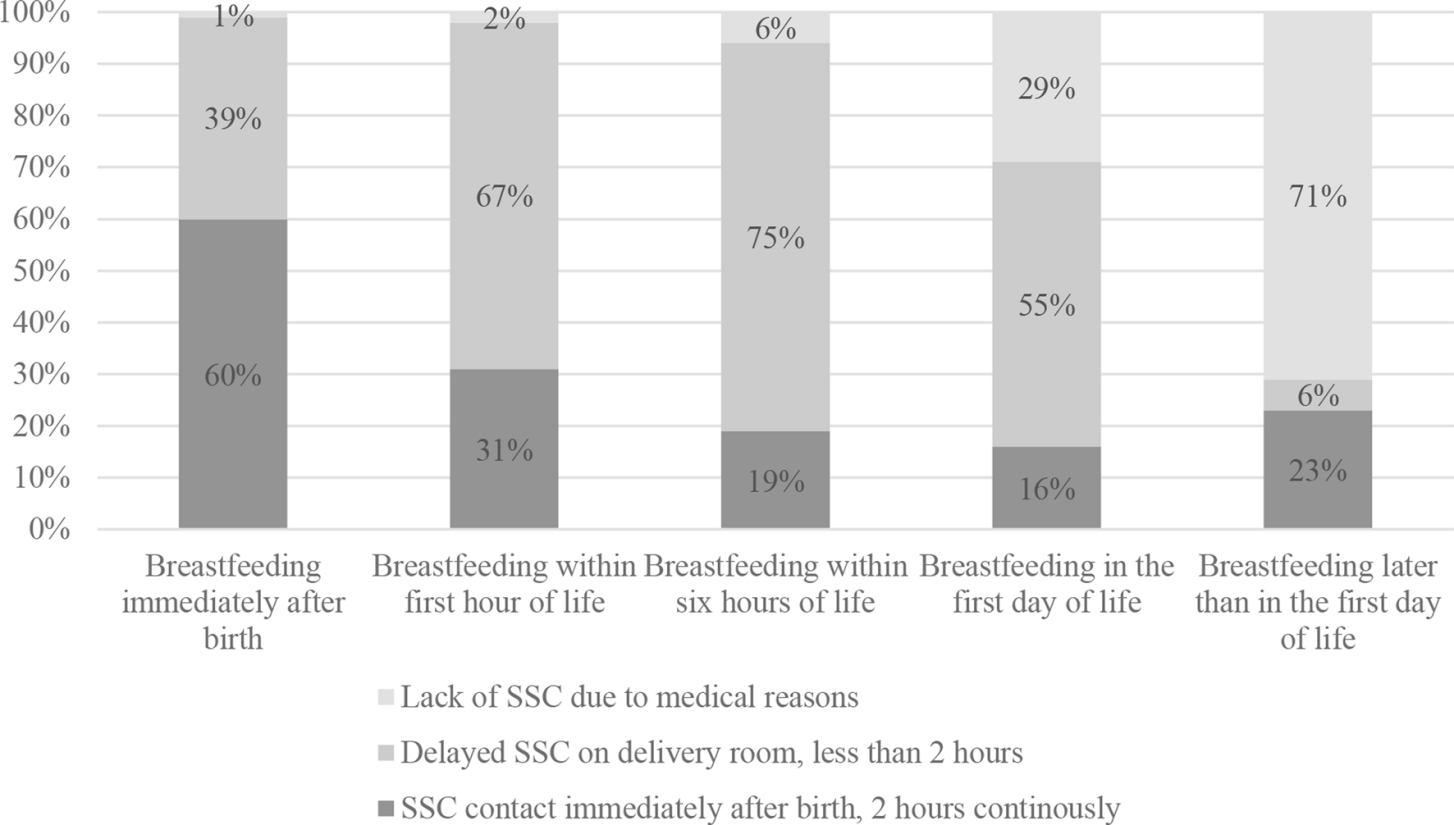
Skin-to-skin contact (SSC) in different groups of newborns, depending on the time of the first breastfeeding

More than half of the refugee mothers (*n* = 353; 52%) breastfed their newborn immediately after birth, and nearly one in five women (*n* = 131; 19%) within the first hour of life. Delayed first breastfeeding concerned above 21% (*n* = 147) of mothers of which 12% (*n* = 79) put the baby into the breast within 6 h after delivery, 7% on the first day (*n* = 51), meanwhile 2% (*n* = 17) on the second day postpartum. In the case of 8% of women, breastfeeding was not possible in the postpartum period because of medical reasons of the newborn (*n* = 52). A relationship between the timing of the first breastfeeding and SSC has been observed (Fig. 3).

In the group of mothers able to breastfeed immediately after delivery^2^ (*n* = 353), the majority were those who experienced SSC promptly for 2 h without interruption (*n* = 208; 60%), and 39% of this group (*n* = 138) had SSC in the delivery room but shorter than the recommended 2 h. In a group of mothers who breastfed their babies within the first hour of life (*n* = 131), 31% (*n* = 41) experienced SSC contact consistent with best practices, and 67% experienced delayed SCC lasting less than 2 h. Regarding mothers who breastfed their babies within the first six hours of birth (*n* = 79), 6% (*n* = 5) did not experience SSC, and in the case of 75% (*n* = 59), SSC was delayed and lasted less than 2 h. In the group of mothers who breastfed with delay but still managed to put the baby to the breast on the first day of the baby’s life (*n* = 51), the percentage of women with SSC was more than half (*n* = 28; 55%). Optimal SSC was experienced by only 16% of mother-baby dyad (*n* = 8) in this group, and about one third of mother did not experience SSC with their baby at all. The lack of SSC was associated with a delay in the first breastfeeding. Among mothers who did not breastfeed until the second day postpartum (*n* = 17), a majority had no SSC (*n* = 12; 71%).

## Mode of feeding before and after lactational counseling

**Fig. 4:**
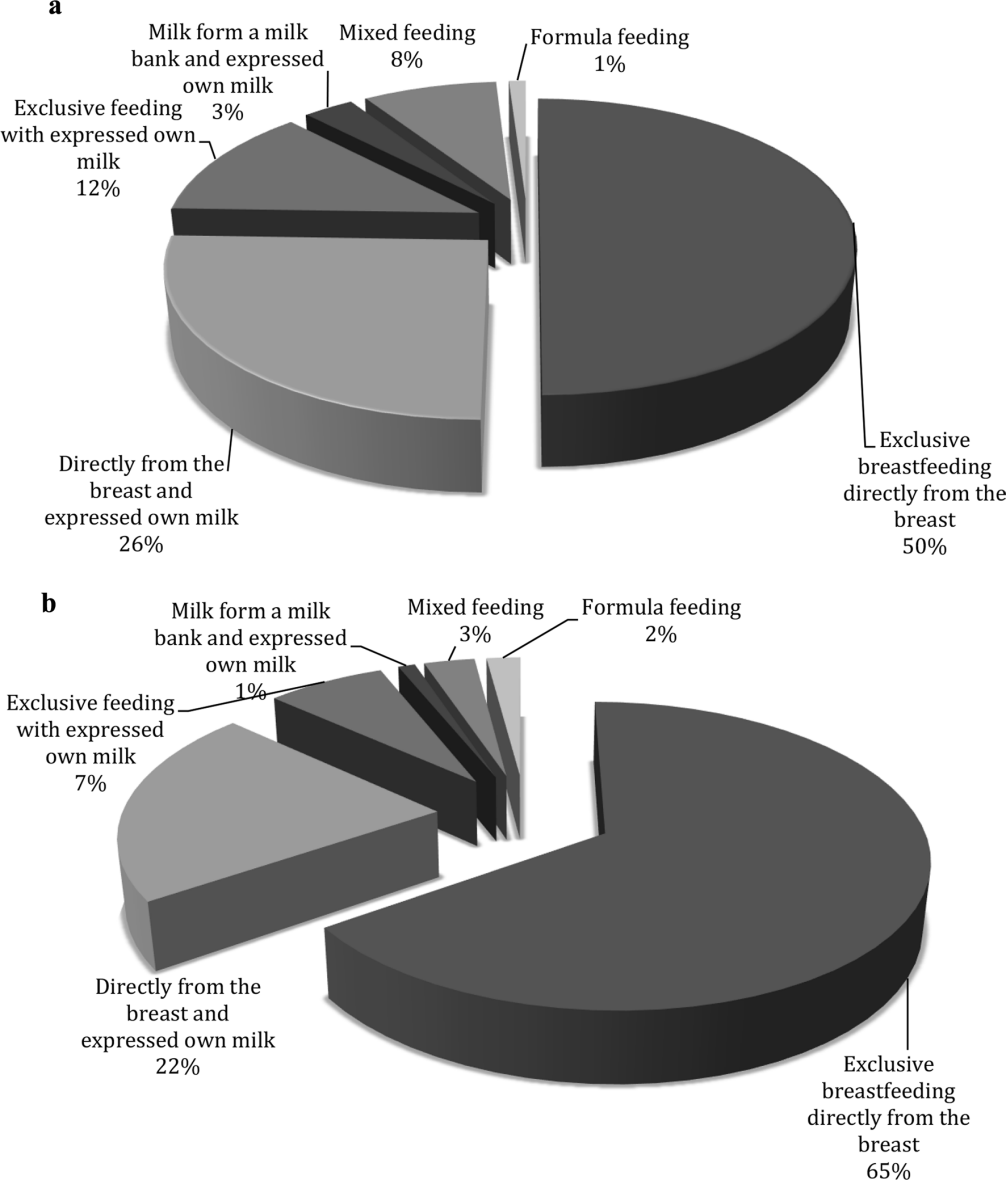
**a** Mode of feeding before lactational counseling (*n* = 98). **b** Mode of feeding after lactational counseling (*n* = 98)

The 98 mothers who had lactation consultations required continued lactation support after the time had passed. Among mothers who needed follow-up visits, 89 mothers were breastfeeding during their first lactation counseling, 7 among them (7%) were mixed feeding and one was feeding with formula (1%) (Fig. 4a). Breastfeeding mothers included mothers who fed exclusively directly from the breast, mothers who fed directly from the breast and with expressed own milk, mothers who fed only with expressed own milk, and mothers who fed with their own expressed milk supplemented by donor human milk. Detailed information on changes in infant feeding in the course of the counseling given is presented in Table 2.

**Table 2:** Changes in infant feeding in the course of the counseling given in a group of refugee mothers from Ukraine

Before lactation counseling	After completion of lactation counseling	*N*
Exclusive breastfeeding directly from the breast	• Exclusive breastfeeding directly from the breast	43
	• Directly from the breast and expressed own milk	6
Directly from the breast and expressed milk	• Exclusive breastfeeding directly from the breast	14
	• Directly from the breast and expressed own milk	9
	• Exclusive feeding with expressed own milk	1
	• Formula feeding	1
Exclusive feeding with expressed milk	• Exclusive breastfeeding directly from the breast	5
	• Directly from the breast and expressed own milk	4
	• Exclusive feeding with expressed own milk	3
Milk from a milk bank and expressed milk	• Milk from a milk bank and expressed milk	1
	• Exclusive feeding with expressed milk	1
	• Formula feeding	1
Mixed feeding: breastfeeding directly from the breast and Formula feeding	• Exclusive breastfeeding directly from the breast	2
	• Mixed feeding: expressed own milk and formula feeding	1
Mixed feeding: breastfeeding directly from the breast, expressed milk and formula feeding	• Directly from the breast and expressed own milk	2
	• Exclusive feeding with expressed own milk	1
Mixed feeding: expressed milk and formula feeding	• Breastfeeding directly from the breast and formula feeding	1
	• Exclusive feeding with expressed own milk	1
Formula feeding	• Breastfeeding directly from the breast and formula feeding	1

In the course of the lactation counseling in the group of breastfeeding mothers, 91% (89 out of 98) managed to continue breastfeeding in the chosen mode (Fig. 4b). The percentage of mothers exclusively breastfeeding directly from the breast increased from 50% (*n* = 49) to 65% (*n* = 64). The percentage of mothers feeding directly from the breast and feeding with expressed milk decreased from 26% (*n* = 25) to 21% (*n* = 21) mostly because many mothers switched to breastfeeding directly from the breast. The percentage of mothers feeding exclusively with expressed own milk decreased from 12% (*n* = 12) to 7% (*n* = 7) mostly because mothers changed their way of feeding into breastfeeding directly from the breast or feeding directly from the breast and with expressed own mother milk given in a different way. The percentage of children fed by expressed own mothers milk supplemented with donor milk decreased due to the hospital discharge (in Poland donor milk is given only for hospitalized babies). The percentage of children fed mixed decreased from 8% (*n* = 8) to 3% (*n* = 3) and thanks to breastfeeding counseling, the mothers switched to exclusive breastfeeding directly from the breast or to exclusive feeding with expressed own milk. One mother feeding exclusively with formula managed to stimulate lactation and switch to mixed feeding. However, two mothers who breastfed in the chosen mode couldn’t manage to maintain lactation. Thus, the percentage of children fed with formula increased from 1 (*n* = 1) to 2%, (*n* = 2) (Fig. 4b).

## Discussion

Intervention programs for refugee populations seldom provide adequate support for the mental health needs and breastfeeding challenges of displaced women (Summers and Bilukha 2018; Diwakar et al. 2019; Jakobsen et al. 2003; Corley 2021; Dall’Oglio et al. 2020; Dybdahl 2001). This results in suboptimal infant and young child feeding practices in refugee populations around the world and is associated with health problems, as well as increased risk of mental disorders in resettled women (Summers and Bilukha 2018; Jakobsen et al. 2003; Corley 2021; Dall’Oglio et al. 2020). While research shows that the relationship between breastfeeding and maternal mental health is complex and bidirectional, a recent study suggests that strengthening breastfeeding confidence during lactation consultations may reflect in the improvement of the woman’s mental health (Bugaeva et al. 2023; Pezley et al. 2022).

The Guidelines and Recommendations for Infant and Young Child Nutrition in Emergencies established by the Global Nutrition Cluster, consisting of several partners, and led by the United Nations Children’s Fund (UNICEF), recommend many proven interventions to properly feed refugee children. The most important activities include implementing hospital practices supporting breastfeeding, creating baby-friendly spaces free from aggressive marketing of breast milk substitutes, and providing mothers with qualified counseling and training of health care workers (WHO 2003; IFE Core Group 2017). Despite the growing number of emergencies around the world, these interventions are still not widely implemented, and their effects are not easy to assess (Dall’Oglio et al. 2020; Iellamo et al. 2024).

Numerous studies have documented the positive influence of certain hospital practices on breastfeeding outcomes (Van Dellen et al. 2019). Among these practices, SSC has emerged as a pivotal factor that can significantly improve breastfeeding success rates (Lau et al. 2018; Karimi et al. 2019). Over 71% of refugee newborns were breastfed early after birth in hospitals involved in emergency response in Poland. It is noteworthy that almost all units providing support were at a high level of lactational care standard. They are participating in the governmental program to increase access to HM for vulnerable infants, including the provision of donor milk (Pro-Life Program, National Health Program, Aim: Increase access to human milk by establishing a network of human milk banks in Poland) (Karimi et al. 2019). However, all hospitals in Poland face problems with lactation and psychology specialists due to a lack of adequate funding. Hospital practices that support the onset of lactation, such as SSC and early latch-on of the breast as well as giving *colostrum* as soon as possible for the first feeding, are legally guaranteed in Poland by a decree of the Minister of Health on Organizational Standard of Perinatal Care. The percentage of mothers who did not experience SSC was negligibly low in the group of women who early breastfed their babies (1%). Delayed SSC was associated with postponed first breastfeeding, which was particularly evident when putting babies into the breast was not done immediately, but in the first hour or first six hours of the life. Lack of SSC was associated with delayed breastfeeding as far as the first day postpartum and beyond. This is consistent with the systematic review and meta-analysis findings (Karimi et al. 2019). We observed that in the study population, the applied SSC significantly improved the rate of early breastfeeding in comparison to available data concerning the Ukrainian population (71% vs. 65%).

In the current project, mothers from Ukraine received multidimensional support with no cost and no referral from other healthcare professionals thanks to the UNICEF Refugee Response Office in Poland to ensure proper nutrition for children. Our results revealed that most women who come in for lactation counseling need one-time support (66% of all counseling given). Among those who needed to follow up in lactation counseling, the majority were breastfeeding at the time of requesting but were experiencing difficulties that were bothering them. Most mothers who experienced problems with lactation and needed a second visit to a lactation consultant managed to maintain breastfeeding directly from the breast while limiting the use of expressed own milk.

In this study we did not obtain detailed data on the place of residence of refugee mothers, nor was a detailed interview conducted on their war and refugee experiences, but based on the available literature (Chrzan-Dentkoś et al. 2022), we imagine that the loss of previous lifestyle and safety, separation from partner/husband (most women were traveling alone or with their children), the necessity to adapt to a new country, socioeconomic disadvantage, lack of knowledge of the Polish language and fear concerning relatives who stayed in Ukraine caused them to experience severe stress, which could have an impact on pregnancy outcomes and problems with breastfeeding. Summers and Bilukha (2018), using a survey of internally displaced persons in Ukraine, found that conflict-related stress is the most frequently reported reason for early breastfeeding termination. Moreover, a difficult and long journey in overcrowded means of transport from the previous place of residence, long waiting times at the border crossing, low temperatures, and lack of support from loved ones during the journey. All this could have contributed significantly to the stress evoked in pregnant women and breastfeeding mothers. Even though not all women were probably affected directly by armed conflict exposure with the same intensity, they still can develop symptoms of mental health disorders (Chrzan–Dentkoś et al. 2022).

The requirement for psychological support was relatively low in the group of mothers who attended lactation counseling (*n* = 227, 33%) which contrasts with the results of the intervention study conducted by Chrzan-Dętkoś et al. (2021) who observed that women who seek such professional breastfeeding support show increased severity of mental health difficulties. In our study, in the population of refugee mothers in 99% of cases, no mental disorders such as postpartum depression, PTSD, anxiety disorders, etc. were found. This result may be related to the fact that most mothers, despite the availability of free psychological counseling at 8 of the 11 hospitals, did not undergo a professional diagnosis with a psychologist. Out of the mothers who sought psychological support, only three were referred for further diagnosis of mental disorders. Additionally, there were only a limited number of follow-up visits (Fig. 2). Refugee mothers may be at risk for poor mental health during the postpartum period, however, studies show that they have difficulty meeting their mental health care needs may not exhibit help-seeking behaviors, and do not necessarily receive the care they need even when healthcare is available to them (Sword et al. 2006; Donnelly and McKellin 2007; O’Mahony and Donnelly 2007; Snow et al. 2021; Villagran et al. 2021). Therefore, the low demand for psychological counseling could be caused by this undesirable phenomenon. What’s more, the lack of high demand for psychological counseling may also be related to the fact that many mental disorders become visible much later after delivery (Ayers et al. 2016; Alshikh et al. 2021). Therefore, at the time of the study, mothers may not have been aware of the risk of developing disorders that they are under due to their difficult experiences of being refugees.

In addition, newly resettled mothers are less likely to seek out professional help due to various barriers including stigma and language (Nicholson et al. 1998). Studies show that the language barrier through the resettlement process impedes refugee women’s access to services, which in turn can negatively affect their psychological well-being (Hou and Beiser 2006; Green 2017). The language barrier makes counseling such as psychological counseling particularly difficult, as it requires privacy, which precludes interpreter participation (Krystallidou et al. 2023). Moreover, research shows that refugee mothers in many cases prioritize their children’s needs at the expense of their own well-being (Nicholson et al. 1998). We observed a significantly greater interest of mothers in lactation counseling aimed at overcoming difficulties related to breastfeeding than in psychological counseling. Therefore, the need to provide newborn infants with appropriate nutrition and thus, take care of their healthy and proper development seems to be an important motivation for mothers seeking help in this area.

However, the desire to take care of the newborn infant and maintain lactation was not only beneficial from the point of view of the infant’s development but also to the mother’s mental health. A recent systematic review by Alimi et al. (2022) found an association between breastfeeding and a reduced risk of postpartum depression. Moreover, women who breastfed frequently at 3 months postpartum experienced a greater decline in depressive symptoms over time (Hahn-Holbrook et al. 2013). ​​A recent meta-analysis shows that breastfeeding could be considered a potential preventive measure against PPD and is recommended for general use in postpartum women (Xia et al. 2022).

Referring to good practices in perinatal care for refugee women proposed by Chrzan-Dętkoś et al. (2022), in this humanitarian project, emphasis was placed on ensuring the promotion of breastfeeding and SSC. Moreover, by providing access to a psychologist on the maternal ward and providing equipment for simultaneous translation, we tried to overcome help-seeking barriers. Moreover, in two centers, midwives or lactation consultants were Ukrainian citizens, which additionally facilitated communication and receiving appropriate support by refugee mothers from Ukraine. In this study, the following barriers to breastfeeding reported in previous studies were overcome: pain or discomfort when breastfeeding, difficulty with latching on the breast, concerns with adequate milk supply, lack of professional lactation support, and unaccommodating childcare environments (Almqvist-Tangen et al. 2012; Wallwiener et al. 2016).

Our research clearly shows that breastfeeding support and increased SSC reduce the need for psychological counseling among refugee mothers, which could suggest that their well-being at the time of the project implementation was satisfactory. Focusing on the needs of the child and effective breastfeeding could have distracted the mother from her mental health issues and breastfeeding through a biological mechanism (regulation of stress hormone levels) and psychological mechanism (self-efficacy and positive mother-infant interaction), in many cases could inhibit the development of mental health problems at that time.

Although there is evidence that refugee mothers experience mental health issues, there is a gap in the literature on interventions supporting mental health and adjustment for refugee mothers. Humanitarian interventions rarely provide adequate support for refugee mothers’ specific mental health needs and challenges (Daou 2022). Therefore, the multi-directional support for mothers from Ukraine within the UNICEF Refugee Response Office in Poland support was an important element in filling this gap. The results of this study show the scope of support and needs of refugee mothers, thus emphasizing how important such interventions are and how important comprehensive breastfeeding support is, which can protect many women from experiencing mental disorders. Breastfeeding support should address not only the acute phase of an emergency but also the stage of preparing for unexpected crises and building resources and capacity (IFE Core Group 2022). The higher the rate of exclusive breastfeeding before the crisis, the less damage the emergency will cause to vulnerable populations.

## Limitations

Our research is primarily descriptive, with an emphasis on sharing experiences, documenting actions taken, and providing overall results, with a significant lack of methodological rigor. Moreover, we did not use a control group or randomized sampling. Although we tried to apply common breastfeeding merits (early initiation of breastfeeding, exclusively breastfeeding, child ever breastfed, mix-feeding, receiving formula) and describe the assessment tool we used to collect data, our study has shortcomings in this regard. For example, questions about the introduction of complementary feeding were not included. We also did not follow up results of the mode of feeding of children after the counseling ended.

Moreover, mothers were not subjected to routine screening for mental health problems, only mothers who expressed such a desire or medical personnel noticed disturbing symptoms, were scheduled for psychological counseling. Therefore, unfortunately, we do not have information about how many and how seriously mothers suffered from minor mental health problems. However, even if the mothers were experiencing mental health challenges, they may not have been significant because the hospital staff would likely have intervened by offering psychological counseling. When designing future humanitarian programs, a crucial aspect would be the introduction of screening questionnaires in maternity wards in times of crisis, allowing to determine the mental state of refugee mothers staying there.

## Conclusions and future intervention

Multidirectional support in lactation care is a best practice for ensuring the needs of mothers and their children in the perinatal period and applies to refugee mothers. Breastfeeding offers several health and mental benefits for both, mother and child. With the help of multi-faceted lactation counseling, refugee mothers can achieve their breastfeeding goal, which contributes to their well-being and ensures the safety and proper nutrition of their newborn child. In future humanitarian interventions, it is essential to address the language barrier in psychological assistance by using simultaneous translators. Additionally, mothers need to be encouraged to seek psychological counseling by emphasizing the benefits for them and their children. It is also recommended to include mental disorder screening tests in the recruitment stage to avoid risks to overlook any serious problems that may arise later.

## Electronic supplementary material

Below is the link to the electronic supplementary material.

Supplementary Material 1

## Data Availability

The data that support the findings of this study are available from the corresponding author upon reasonable request and with permission of the Human Milk Bank Foundation.

## Footnotes

1 In Poland, speech-language pathologists deal not only with the correction of speech defects in older children but also with the diagnosis of a short frenulum in infants, which can significantly affect sucking. Therefore, mothers who referred to problems with suckling by the baby were most probably referred to consultations with speech-language pathologists.

2 Immediately after delivery - as soon as possible, before any medical examination, hygiene procedures, measuring and weighing. It is strongly recommended by the WHO even in case of preterm or low-birth-weight infants (WHO recommendations for care of the preterm or low birth weight infant. Geneva: World Health Organization; 2022).
