# Supplementary material for: Good practice in lactation counseling for Ukrainian refugee mothers to ensure the health and mental benefits of breastfeeding – an observational study
Source: Arch Womens Ment Health. 2024 Dec 6;28(2):257–69. doi: 10.1007/s00737-024-01538-x (PMC12018615; doi:10.1007/s00737-024-01538-x)
Supplement: Supplementary file 1 — Supplementary Material 1 [file 737_2024_1538_MOESM1_ESM.docx]

**Appendix 1**

List of hospitals that the refugee mothers from Ukraine were provided with support from lactation consultants, speech-language pathologists, physiotherapists and psychologists within the project conducted by the Human Milk Bank Foundation (Warsaw) on behalf of the UNICEF Refugee Response Office in Poland:

1. Karol Marcinkowski University Hospital in Zielona Góra,
2. Ujastek Medical Center in Krakow,
3. Clinical University Center in Gdańsk,
4. A. Falkiewicza Specialist Hospital in Wrocław,
5. Clinical Provincial Hospital No. 1 F. Chopin in Rzeszów,
6. Independent Public Clinical Hospital No. 1 in Lublin,
7. Pro-Familia Specialist Hospital in Rzeszów,
8. Polish Mother Institute Center in Łódź,
9. Ludwik Rydygier Provincial Polyclinical Hospital in Toruń,
10. University Hospital in Wrocław,
11. MSWiA Hospital in Warsaw.
